# Amino acid transporters implicated in endocytosis of *Buchnera* during symbiont transmission in the pea aphid

**DOI:** 10.1186/s13227-016-0061-7

**Published:** 2016-11-21

**Authors:** Hsiao-ling Lu, Chun-che Chang, Alex C. C. Wilson

**Affiliations:** 1 Department of Biology, University of Miami, Coral Gables, FL 33146 USA; 2 Department of Entomology, College of Bioresources and Agriculture, National Taiwan University, Taipei, 10617 Taiwan; 3 Research Center for Developmental Biology and Regenerative Medicine, National Taiwan University, Taipei, 10617 Taiwan; 4 Institute of Biotechnology, College of Bioresources and Agriculture, National Taiwan University, Taipei, 10617 Taiwan

**Keywords:** Amino acid transporter, Aphid development, Coevolution, Host/symbiont developmental integration, Symbiosis

## Abstract

**Background:**

Many insects host their obligate, maternally transmitted symbiotic bacteria in specialized cells called bacteriocytes. One of the best-studied insect nutritional endosymbioses is that of the aphid and its endosymbiont, *Buchnera aphidicola*. Aphids and *Buchnera* are metabolically and developmentally integrated, but the molecular mechanisms underlying *Buchnera* transmission and coordination with aphid development remain largely unknown. Previous work using electron microscopy to study aphid asexual embryogenesis has revealed that *Buchnera* transmission involves exocytosis from a maternal bacteriocyte followed by endocytotic uptake by a blastula. While the importance of exo- and endocytic cellular processes for symbiont transmission is clear, the molecular mechanisms that regulate these processes are not known. Here, we shed light on the molecular mechanisms that regulate *Buchnera* transmission and developmental integration.

**Results:**

We present the developmental atlas of *ACYPI000536* and *ACYPI008904* mRNAs during asexual embryogenesis in the pea aphid, *Acyrthosiphon pisum*. Immediately before *Buchnera* invasion, transcripts of both genes were detected by whole-mount in situ hybridization in the posterior syncytial nuclei of late blastula embryos. Following *Buchnera* invasion, expression of both genes was identified in the region occupied by *Buchnera* throughout embryogenesis. Notably during *Buchnera* migration, expression of both genes was not concomitant with the entirety of the bacterial mass but rather expression colocalized with *Buchnera* in the anterior region of the bacterial mass. In addition, we found that *ACYPI000536* was expressed in nuclei at the leading edge of the bacterial mass, joining the bacterial mass in subsequent developmental stages. Finally, quantitative reverse transcription real-time PCR suggested that early in development both transcripts were maternally provisioned to embryos.

**Conclusions:**

We venture that *ACYPI000536* and *ACYPI008904* function as nutrient sensors at the site of symbiont invasion to facilitate TOR-pathway-mediated endocytosis of *Buchnera* by the aphid blastula. Our data support earlier reports of bacteriocyte determination involving a two-step recruitment process but suggest that the second wave of recruitment occurs earlier than previously described. Finally, our work highlights that bacteriocyte-enriched amino acid transporter paralogs have additionally been retained to play novel developmental roles in both symbiont recruitment and bacteriome development.

**Electronic supplementary material:**

The online version of this article (doi:10.1186/s13227-016-0061-7) contains supplementary material, which is available to authorized users.

## Background

A compelling challenge in symbiosis research concerns identifying and characterizing the mechanisms of host/symbiont metabolic and developmental integration [1]. Recently, we began to address this challenge by studying host/symbiont developmental integration in the pea aphid *Acyrthosiphon pisum* and its obligate endosymbiont *Buchnera aphidicola* [2]. Like all aphids, *A. pisum* feeds exclusively on plant phloem sap, a diet that is rich in sugar but limited in provision of essential amino acids and vitamins [3–5]. To overcome the nutritional deficiency of their diet, aphids anciently acquired *B. aphidicola*, an endosymbiont that metabolically compensates the nutritional shortcomings of their diet [1, 6, 7]. *Buchnera* is transmitted from generation to generation through transovarial transmission [8], and remarkably aphids cured of *Buchnera* infection fail to reproduce [9–12].

Aphids are cyclical parthenogens, capable of both sexual and asexual modes of reproduction. In asexual aphids, one ovariole contains a series of embryos at various developmental stages [13]. Parthenogenetic embryogenesis has been described by Will [14] and Miura et al. [8]. The parthenogenetic embryo originates from a maturation division of the oocyte by skipping the reduction division of meiosis I, producing a polar body and an oocyte nucleus [8, 15]. The oocyte nucleus is considered to be the incipient parthenogenetic embryo that divides synchronously five times in a common cytoplasm to form the syncytial embryo with 32 syncytial nuclei. Cellularization of the syncytial nuclei then generates the blastula embryo. Primordial germ cells that are specified in the posterior region of the blastula separate the remaining uncellularized syncytial nuclei into two locations: (1) the central syncytium (in the center of the blastoderm) and (2) the posterior syncytium (at the posterior end of the blastoderm) [8].

Developmental integration of *Buchnera* into host aphids starts with transmission of a small population of *Buchnera* from maternal bacteriocytes, the specialized aphid cells accommodating *Buchnera*, to a blastula [8]. Transmission of *Buchnera* involves exocytosis from maternal bacteriocytes followed by endocytosis by the developing blastula. During exocytosis, individual *Buchnera* lose their host-derived symbiosomal membrane (a perisymbiont membrane that encloses individual *Buchnera* cells) and migrate naked across the extracellular space between the maternal bacteriocyte and the posterior pole of the blastula to reacquire their symbiosomal membrane by endocytotic passage into the blastoderm (stage 7 of development) [8, 16]. Following transmission, the *Buchnera* population, together with aphid uncellularized syncytial nuclei, are subdivided into uninucleate bacteriocyte cells [8]. After this, bacteriocyte cells proliferate [17], and maturation of the bacteriome proceeds [2]. Aphid bacteriomes, the organ of symbiosis, comprise two cell types: bacteriocytes that house *Buchnera* and intercalating sheath cells that do not house *Buchnera* [18, 19]. Localization of the transcription factor Distal-less (Dll) reveals that bacteriocytes are recruited through two steps. In the first step, bacteriocyte cells originate from the syncytial nuclei that localize prior to *Buchnera* invasion in the posterior syncytium of the blastoderm embryo [17]. In the second step, bacteriocyte cells originate from approximately 40–60 Dll-expressing cells that migrate across the germband and intercalate between the original bacteriocytes during germband extension [17]. In contrast to the original proposal by Braendle et al. [17] that bacteriocytes are recruited in two steps, Koga et al. [16] hypothesized, on the basis of the smaller nuclei of the second group of cells and their localization between the original bacteriocyte population, that the second group of cells may in fact be sheath cells and not bacteriocytes. In late embryogenesis, aphid embryos undergo katatrepsis when the head is repositioned from the posterior to the anterior pole of the egg chamber resulting in inversion of the body axis; during this maturation, bacteriocyte cells are positioned to the dorsal side of the embryo where they later form the mature bacteriome [2, 8, 16].

Work to date in asexually reproducing *A. pisum* has demonstrated that specification of bacteriocytes occurs independently of symbiont invasion from mother to progeny [17] and that *Buchnera* transmission involves exo- and endocytotic cellular processes [16]. Bacteriocytes are widely found in sap-feeding and blood-feeding insect lineages, and yet little is known about the molecular network underlying bacteriocyte morphogenesis or the mechanisms that regulate transmission and developmental integration of symbionts.

Here, by studying the expression of two aphid amino acid transporter mRNAs during asexual embryogenesis, we gain insight into the molecular mechanisms that regulate transmission and developmental integration of *Buchnera* into aphid hosts. Amino acid transporters are the membrane proteins responsible for cellular nutrient exchange and nutrient sensing [20]. As nutrient sensors, amino acid transporters act as transceptors: dual-function molecules that both transport amino acids and function as receptors sensing extracellular amino acid availability and modulating intracellular nutrient-responsive signaling pathways [20–23]. Amino acid transporters have been implicated in a broad diversity of cellular and developmental functions that include control of cell growth [24–27], cell proliferation [28–30], regulation of translational capacity [31], lysosome biogenesis [32], and coordination of endocytosis/phagocytosis [26, 33]. The *A. pisum* genome encodes 40 putative amino acid polyamine organocation superfamily amino acid transporters, eight of which are expressed in bacteriocyte cells [34]. So far, bacteriocyte-enriched amino acid transporters have been implicated in regulating host and symbiont mediated amino acid biosynthesis in bacteriocytes [35], and comparative work has demonstrated that retention of duplicated amino acid transporters can result from selection to function in symbiosis [36–38]. Within *A. pisum* bacteriocytes, the two amino acid transporters that are most highly expressed are the eukaryotic-specific amino acid/auxin permease (AAAP) family (TC# 2.A.18) member, *ACYPI000536*, and the amino acid/polyamine/organocation (APC) family (TC# 2.A.3) member, *ACYPI008904* [34]. *ACYPI000536* and *ACYPI008904* are gene identifiers that have been assigned in the *ACY* *rthosiphon* *PI* *sum* reference genome available at AphidBase [39]. To enhance readability in this paper, we will refer to *ACYPI000536* as *AAAP*-*536* and *ACYPI008904* as *APC*-*8904*. Using whole-mount in situ hybridization and quantitative PCR, we present the developmental atlas of *AAAP*-*536* and *APC*-*8904* mRNAs during asexual embryogenesis. In interpreting the developmental atlas of *AAAP*-*536* and *APC*-*8904* expression, we propose that these amino acid transporters play novel roles in endocytosis of *Buchnera* by aphid blastula and in bacteriocyte development during asexual aphid embryogenesis.

## Methods

### Aphids

*Acyrthosiphon pisum* line LSR1 [40] and a Taiwanese line NTU [41] were maintained on *Vicia faba* and incubated at 20 °C under a 16-h light/8-h dark cycle. Oocytes and embryos for whole-mount in situ hybridization were dissected from wingless adult females in phosphate-buffered saline (PBS; 10 mM phosphate buffer, 154 mM NaCl, pH 7.4; Sigma-Aldrich). Oogenesis and embryogenesis staging was according to Miura et al. [8] and germ cell locations according to Chang et al. [42].

### Cloning of *AAAP*-*536* and *APC*-*8904* gene fragments

Primer pairs for amplifying *AAAP*-*536* and *APC*-*8904* gene fragments were designed via MacVector version 7.2.2 (Accelrys Inc.) according to the gene sequences in AphidBase 2.1 [39]. Primers designed to *AAAP*-*536* coding sequence were further checked using Primer-BLAST against the pea aphid genome Acyr_2.0 assembly, confirming that each primer hit only its isoform targets (amplicon located at 1719–2387 of XM_008185850.2 and 1150–1818 of NM_001246304.1). Due to the high similarity of *APC*-*8904* nucleotide sequence to that of its paralogs: 82.56% coding sequence similarity to *ACYPI008323* and 76.43% coding sequence similarity to *ACYPI002633*, we designed *APC*-*8904* gene-specific primers for hybridization to the 3′ untranslated region (UTR) of *APC*-*8904.* Nucleotide similarity in the 3′ UTR of *APC*-*8904* and its paralogs is much lower such that similarity between *APC*-*8904* and *ACYPI008323* is 66.32% across the 193 base pairs (bp) of alignable 3′ UTR sequence and that between *APC*-*8904* and *ACYPI002633* is 37.99% across the 766 bp of alignable 3′ UTR sequence. For *AAAP*-*536*, 669 bp of open reading frame (ORF) was amplified from a plasmid containing the gene’s full-length cDNA [35] with forward primer 5′-TGGGATGCTAAGTATTCACTTCC-3′ and reverse primer 5′-TTCAATGGTAGAATTTATAGTGT-3′. For *APC*-*8904*, 566 bp of the 3′UTR was amplified from whole insect LSR1 cDNA [35] with forward primer 5′-TCCTCCCCTATGTTTCCACG-3′ and reverse primer 5′-TTCTCAGCGAAGACACACCGTC-3′. Amplification of gene fragments was performed starting with an initial denaturation at 94 °C for 30 s, followed by 30 cycles at 94 °C for 10 s, 50 °C for 30 s, 72 °C for 1 min and a final extension at 72 °C for 5 min. Both amplicons were subcloned into pGEM-T vector (Promega), and the insert sequences and orientations were confirmed by DNA sequencing before RNA probe synthesis.

### Whole-mount in situ hybridization and microscopy

Hybridizations in situ with digoxigenin (DIG)-labeled RNA probes to whole-mount ovaries were performed as described by Chang et al. [43]. The antisense and sense DIG-labeled RNA probes were transcribed with SP6 or T7 RNA polymerase (New England Biolabs). Transcription was carried out from 1 μg linearized plasmid DNA in 20 μl reaction mixtures containing 2 μl 10 × RNA polymerase reaction buffer (400 mM Tris–HCl, 60 mM MgCl_2_, 20 mM spermidine, 10 mM dithiothreitol, pH 7.9; New England Biolab), 2 μl 10 × DIG RNA labeling mix (Roche), 0.5 μl RNAse inhibitor (Invitrogen), and 2 μl RNA polymerase (New England Biolabs). The reaction mixtures were incubated for 4 h at 37 °C, and the products were verified by size on a 1.5% agarose gel containing 2.2 M formaldehyde. After adding sodium acetate to 0.3 M, nucleic acids were precipitated with ethanol until further use.

Dissected ovaries from *A. pisum* line LSR1 containing developing oocytes and embryos were fixed in 3.8% formaldehyde (VWR) in PBS at 4 °C overnight. Each experiment included antisense riboprobe (experimental group) and sense riboprobe (control group) treatments, and the working concentration for each riboprobe was 2.0 ng/μl. Other steps followed the protocol of Chang et al. [43]. Probe hybridizations for *AAAP*-*536* and *APC*-*8904* were carried out at 68 and 60 °C, respectively, and specimens were incubated overnight at 4 °C with anti-DIG Fab fragments conjugated to alkaline phosphatase (Roche), diluted 1:500 in 1× blocking reagent (Roche). Nitroblue tetrazolium (NBT)/5-bromo-4-chloro-3-indolyl phosphate (BCIP) (Roche) was applied to detect the hybridized probes. We terminated the substrate reaction concurrently for experimental (antisense riboprobe) and control (sense riboprobe) samples based on the relative intensity of signal in the region of embryos occupied by *Buchnera* in the antisense riboprobe (experimental) and sense riboprobe (control) treatments. Following signal development, samples were counterstained with 2 μg/ml of 4′,6-diamidino-2-phenylindole (DAPI) (Sigma-Aldrich) at room temperature for 1 h and mounted in 70% glycerol (Sigma-Aldrich) in PBS at 4 °C overnight. Photographs of NBT/BCIP staining were acquired with a Zeiss Axiovert 200 microscope connected to a Zeiss AxioCam ICc1 camera. Photographs of DAPI staining were taken with the Leica SP5 laser scanning confocal microscope in the University of Miami, Department of Biology Microscopy Core Facility. The experiment was performed 13 times for *AAAP*-*536* and 6 times for *APC*-*8904*. Samples collected from each developmental stage were counted to report on the repeatability of our results; count data are available in Additional file 1: Table S1 for *AAAP*-*536* and Additional file 2: Table S2 for *APC*-*8904*.

### Real-time PCR

Total RNA for each biological replicate was prepared from asexual adult female ovarioles (equivalent to 600–700 ovarioles) from *A. pisum* line NTU. Each ovariole contains germaria, oocytes and developing embryos at various stages of development enveloped within egg chambers. Dissection was performed under a Leica EZ4 Stereo Microscope with non-removable 16× eyepieces at 40× magnification on a 12-well watch glass with one individual adult dissected into each well. To avoid contamination, ovaries were separated from other somatic tissues and the somatic tissues were removed using a 200-μl pipetman (Eppendorf). Ovaries were washed several times with ice-cold PBS until no maternal bacteriocytes or other somatic tissue remained in the well. Washes were performed with great care to avoid damaging the ovary structure. Under 56× magnification on the Leica Stereo Microscope, using a black enameled pin (diameter 0.25 mm; Entomoravia), the egg chambers were separated on the follicular stalk between embryos that were stage 5 or younger and stage 6 or older. Importantly, at developmental stage 6, embryos are clearly identifiable morphologically by the appearance of their primordial germ cells and enlarged follicle cells [8]. Two sets of embryos were collected: set A that included germaria and stage 1 to stage 5 embryos and set B that included stage 6 to stage 20 embryos. Total RNA was extracted from embryo sets using a RNeasy Mini kit (Qiagen). To ensure that total RNA was free of DNA, a DNase I digestion was performed twice during RNA purification with the RNase-Free DNase Set (Qiagen). The first treatment was an on-column digestion, and DNase was removed in subsequent wash steps. The second treatment was performed after RNA purification by an in-solution digestion prior to RNA cleanup. Reaction setup followed the manufacturer’s instructions (Qiagen). Total RNA was quantified using an Eppendorf Biophotometer spectrophotometer. Two hundred nanograms of total RNA was reverse-transcribed into cDNA with QuantiTect Reverse Transcription kit (Qiagen) in standard 20 μl reaction mixture. All experiments were performed with three independent biological replicates; RNA for each biological replicate was prepared using an individual set of dissected embryos from the NTU *A. pisum* line.

Primer pairs used to detect gene expression as well as amplicon sizes and amplification efficiencies are given in Additional file 3: Table S3. Ten microliter PCR mixture contained 1× QuantiFast SYBR Green PCR Master Mix (Qiagen), 200 nM of forward primer, 200 nM of reverse primer, and cDNA derived from 9.2 ng of total RNA from set A or set B tissue samples. Each sample was amplified using triplicate technical replicates. Each plate included no reverse transcription controls and no template controls for each primer set. Samples were run on a Rotor-Gene Q real-time PCR cycler (Qiagen). The thermal cycling conditions were started by activating the enzyme reaction with 5 min at 95 °C followed by 40 cycles of 95 °C for 15 s, 52 °C for 15 s, and 60 °C for 20 s. A final melt-curve step was included post-PCR to confirm the absence of any non-specific amplification using Rotor-Gene Q series software (version 1.7). We used the programs geNorm [44, 45], Normfinder [46], and BestKeeper [47] to assess the stability of five control genes (*GAPDH* [34], *EF1α* [48], *RPL7* [49], *RPL32* [50], and *βTUB* [50]) between the samples of set A and set B. Based on the gene stability analyses, *GAPDH*, *EF1α*, and *RPL32* were selected as endogenous controls. Gene expression was compared to embryos before stage 5 using 2^−∆∆CT^ methodology [51] and normalized to the geometric mean of *GAPDH*, *EF1α*, and *RPL32* [45].

### Cytological staining

Dissected ovaries from *A. pisum* line NTU containing developing oocytes and embryos were fixed in 4% paraformaldehyde (Sigma-Aldrich) in PBS at room temperature for 20 min. Following fixation, ovaries were washed with 0.2% of Triton-X 100 (Sigma-Aldrich) in PBS three times, 10 min for each wash. Ovaries were then incubated with 2 μg/ml of DAPI (Sigma-Aldrich) at room temperature for 1 h and mounted in 70% glycerol (Sigma-Aldrich) in PBS at 4 °C overnight. Photographs of DAPI staining were taken with the Leica SP5 laser scanning confocal microscope at National Taiwan University.

## Results

### The developmental atlas of *AAAP*-*536* and *APC*-*8904* expression with reference to the symbiotic bacterial mass

**Fig. 1:**
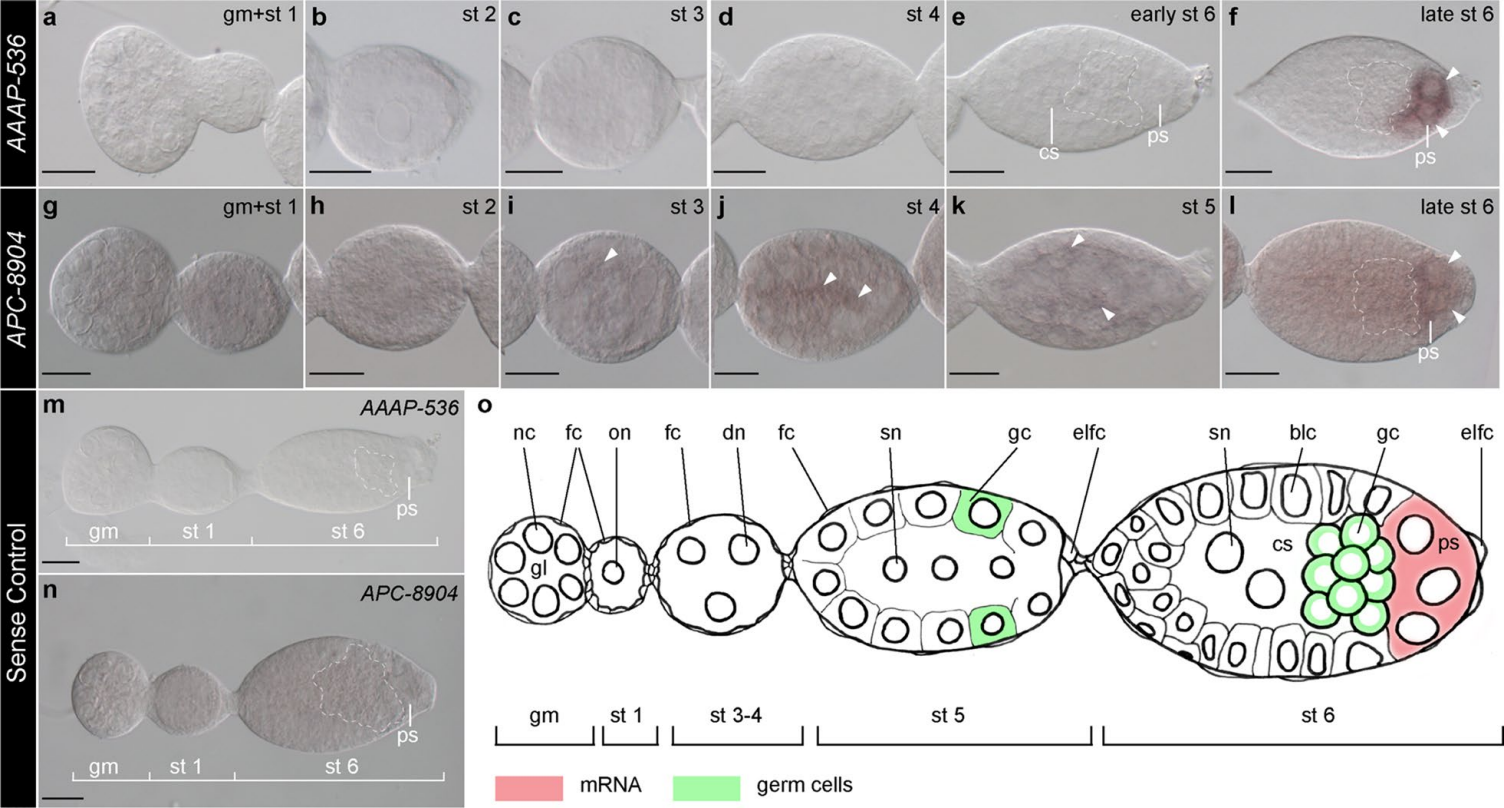
Expression atlas of *AAAP*-*536* and *APC*-*8904* genes in asexual *A. pisum* before symbiont invasion. Staged embryos hybridized with DIG-labeled antisense (**a**–**l**) and sense riboprobes (**m**, **n**) of *AAAP*-*536* and *APC*-*8904*. Signals were revealed by NBT/BCIP brown precipitates. All embryos are displayed with anterior of the germaria to the *left*. *Arrowheads* mark *AAAP*-*536-* and *APC*-*8904*-positive signals. *Dashed lines* indicate the location of germ cells. **a**–**f** Germarium, oocyte, and embryos before *Buchnera* invasion; transcripts of *AAAP*-*536* were detected in the posterior syncytium (ps) of late stage 6 embryo. **g**–**l** Germarium, oocyte, and embryos before *Buchnera* invasion; preferential expression of *APC*-*8904* was detected in the posterior syncytium (ps) of late stage 6 embryo. **m, n** Oocytes and developing embryos hybridized with sense riboprobes. No signal was detected with *AAAP*-*536* sense riboprobes, but ubiquitous background signals were detected with *APC*-*8904* sense riboprobes. **o** Illustration displaying presented developmental stages. Germ cells and specific signals of *AAAP*-*536* and *APC*-*8904* detected in the posterior syncytium by in situ hybridization are indicated by the *color key*. *Scale bars* are all 20 μm. *blc* blastodermal cells, *cs* central syncytium, *dn* dividing nucleus, *eflc* enlarged follicle cells, *fc* follicle cells, *gc* germ cells, *gl* germarial lumen, *gm* germarium, *nc* nurse cells, *on* oocyte nucleus, *ps* posterior syncytium, *sn* syncytial nucleus, *st* stage

**Fig. 2:**
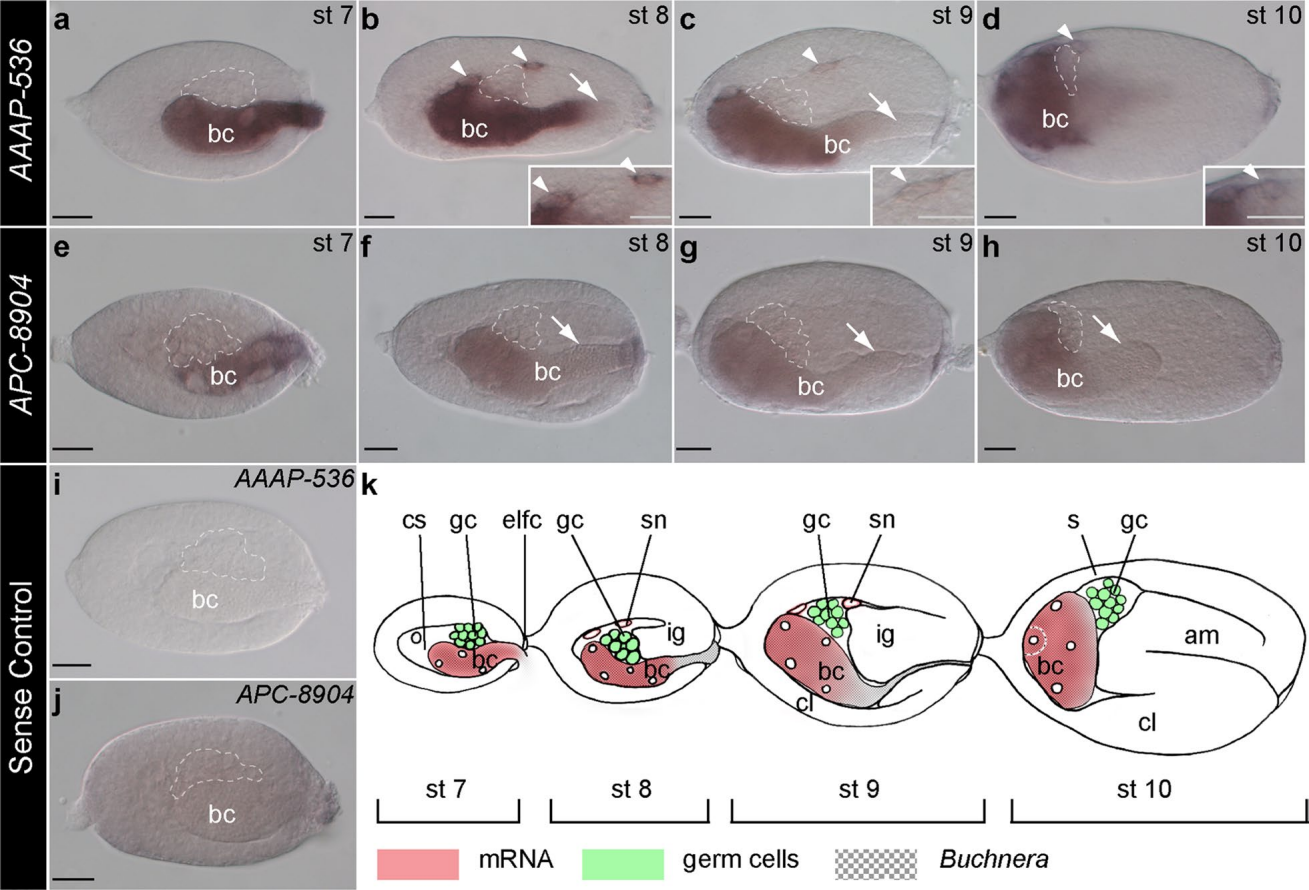
Expression atlas of *AAAP*-*536* and *APC*-*8904* genes in asexual *A. pisum* during symbiont invasion. Staged embryos hybridized with DIG-labeled antisense (**a**–**h**) and sense riboprobes (**i**, **j**) of *AAAP*-*536* and *APC*-*8904*. Signals were revealed by NBT/BCIP brown precipitates. All embryos are displayed with anterior of the germaria to the *left*. *Arrowheads* mark *AAAP*-*536*-positive signals outside of the bacterial mass. *Dashed lines* indicate the location of germ cells. *Arrows* indicate regions of endosymbiotic bacterial mass that are not concomitant with *AAAP*-*536* and *APC*-*8904* signals. **a**–**d** Incorporation of the maternal endosymbiotic bacteria (stage 7), germband invagination (stages 8–9), and germband bending (stage 10). Notably at stages 8–9 most transcripts of *AAAP*-*536* were detected in the anterior region of the bacterial mass. *Insets* in **b**–**d** are magnifications of the *AAAP*-*536* transcripts detected outside the bacterial mass (*arrowheads*). **e**–**h** Expression of *APC*-*8904* was detected in the bacterial region such that the intensity of the signal was strongest in the anterior region of the bacterial mass (stages 8–10). **i, j** Stage 7 embryos hybridized with sense riboprobes. No signal was detected with *AAAP*-*536* sense riboprobes, but ubiquitous signals were detected with *APC*-*8904* sense riboprobes. **k** Illustration displaying presented developmental stages. In situ signals of *AAAP*-*536* and *APC*-*8904*, location of germ cells, and location of *Buchnera* are indicated by the *color key*. *Scale bars* are all 20 μm. *am* amnion, *bc Buchnera* symbionts, *cl* cephalic lobe, *cs* central syncytium, *eflc* enlarged follicle cells, *gc* germ cells, *ig* invaginating germband, *s* serosa, *sn* syncytial nucleus, *st* stage

**Fig. 3:**
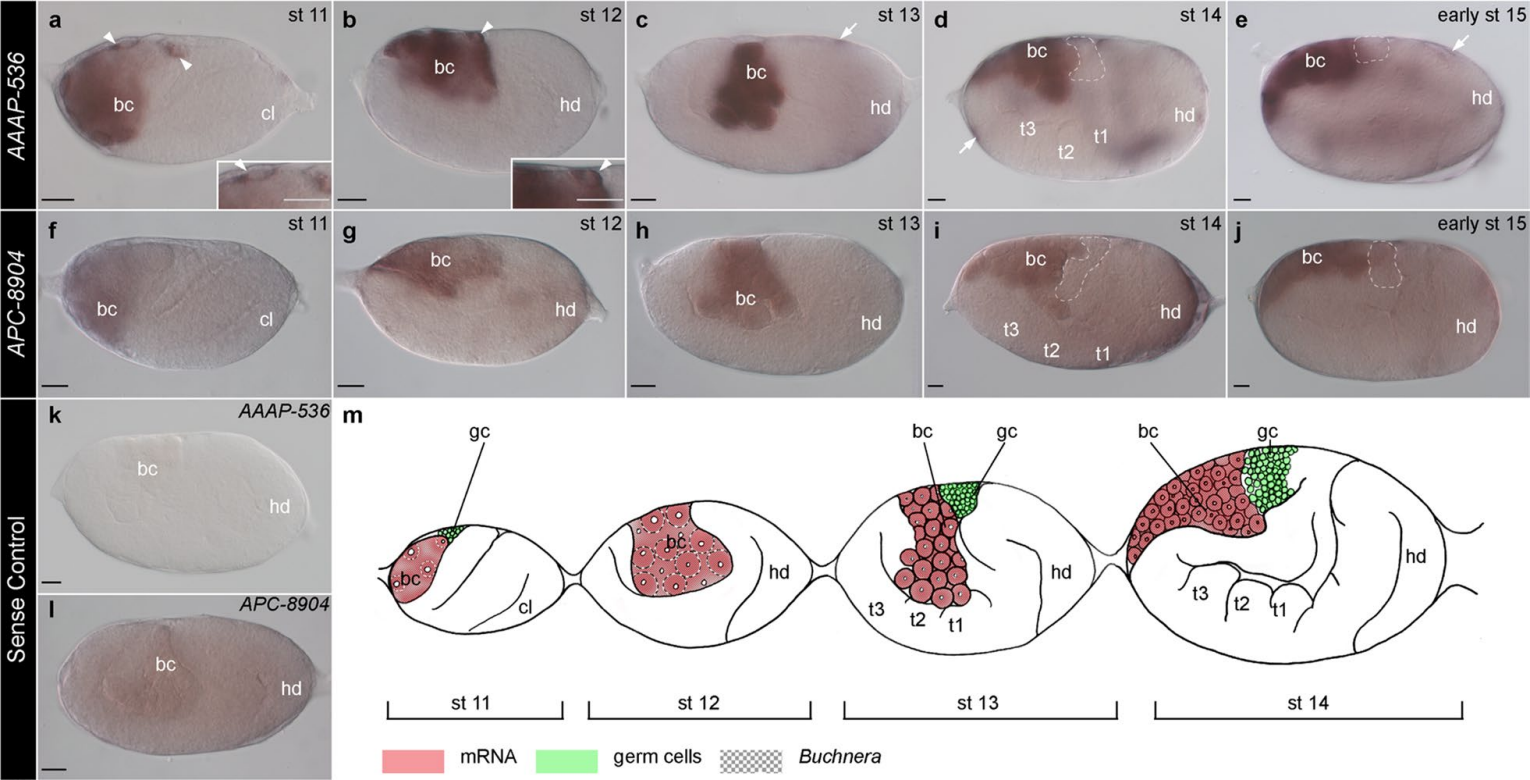
Expression atlas of *AAAP*-*536* and *APC*-*8904* genes in asexual *A. pisum* during bacteriocyte formation. Staged embryos hybridized with DIG-labeled antisense (**a**–**j**) and sense riboprobes (**k**, **l**) of *AAAP*-*536* and *APC*-*8904*. Signals were revealed by NBT/BCIP brown precipitates. All embryos are displayed with anterior of the germaria to the *left*. The cephalic lob (cl) and head (hd) of germband are indicated in the posterior of the egg chamber to the *right*. *Arrowheads* mark relatively strong *AAAP*-*536*-positive signals in **a**, **b**. *Dashed lines* indicate the location of germ cells. *Arrows* indicate *AAAP*-*536* signals detected in the follicle cells in **c**–**e**. **a**–**e** S-shaped embryo (stage 11), twisting embryo (stage 12), limb bud formation (stages 13–14), and flip embryo (stage 15); transcripts of *AAAP*-*536* were detected in the bacterial region at all stages shown. *Insets* in **a**, **b** are magnifications of the relatively strong signals of *AAAP*-*536* (*arrowheads*). **f**–**j** Expression of *APC*-*8904* was detected in the bacterial region. **k, l** Stage 13 embryos hybridized with sense riboprobes. No signal was detected with *AAAP*-*536* sense riboprobes, but ubiquitous signals were detected with *APC*-*8904* sense riboprobes. **m** Illustration displaying presented developmental stages. In situ signals of *AAAP*-*536* and *APC*-*8904*, location of germ cells, and location of *Buchnera* are indicated by the *color key*. *Scale bars* are all 20 μm. *bc Buchnera* symbionts, *cl* cephalic lobe, *gc* germ cells, *hd* head, *st* stage, *t1*–*3* thoracic segments

**Fig. 4:**
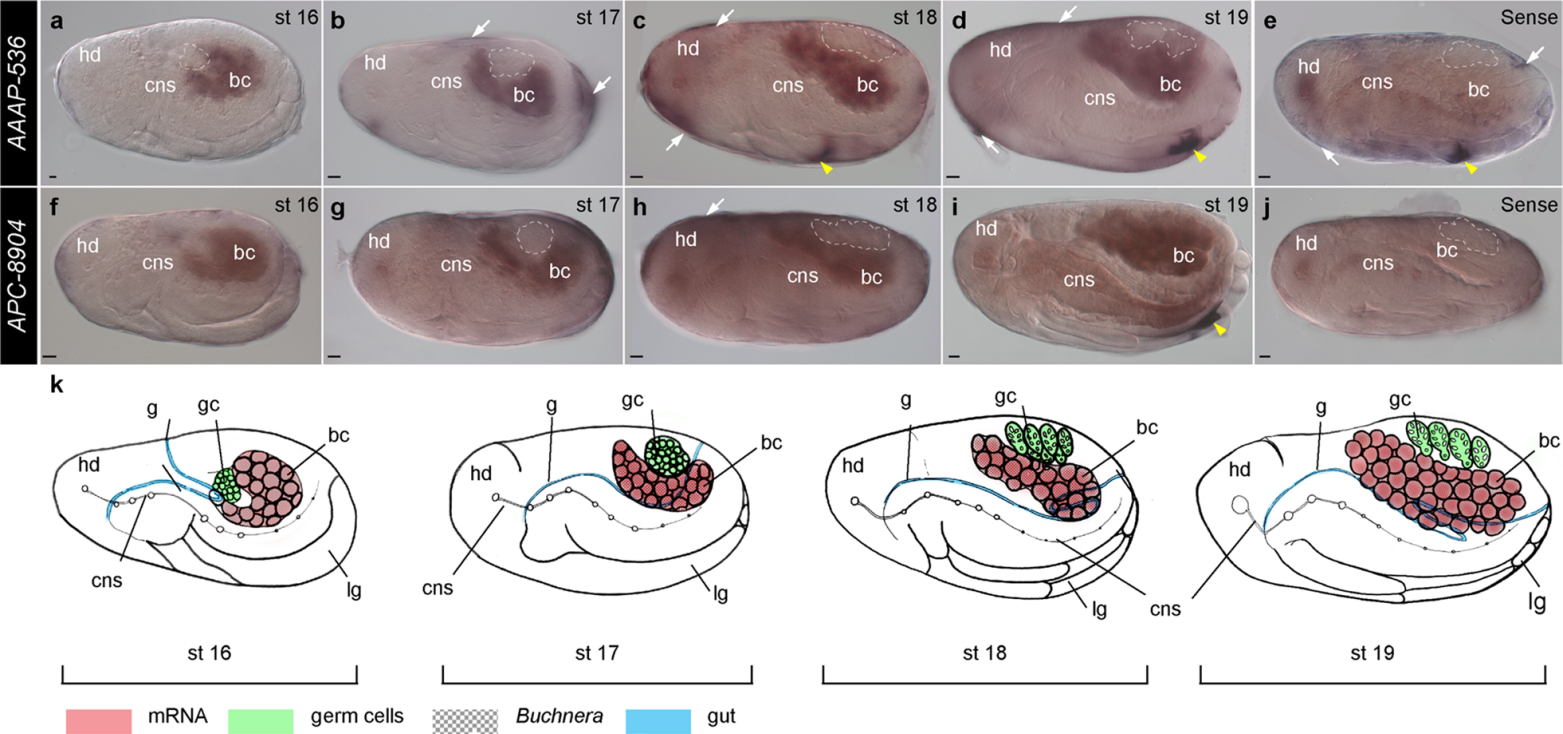
Expression atlas of *AAAP*-*536* and *APC*-*8904* genes in asexual *A. pisum* during bacteriome maturation. Staged embryos hybridized with DIG-labeled antisense (**a**–**d**, **f**–**i**) and sense riboprobes (**e**, **j**) of *AAAP*-*536* and *APC*-*8904*. Signals were revealed by NBT/BCIP brown precipitates. All embryos are displayed with anterior of the germaria to the *left*. *Dashed lines* indicate the location of germ cells. *Arrows* indicate signals detected in the follicle cells and *yellow arrowheads* mark non-specific signals. **a**–**d** Post-flip embryo (stage 16), germband retraction (stages 17–18), and muscle formation (stage 19); transcripts of *AAAP*-*536* were detected in the bacterial region at all developmental stages. **f**–**i** Expression of *APC*-*8904* was detected in the bacterial region. **e, j** Stage 18 embryos hybridized with sense riboprobes. Non-specific signals of *AAAP*-*536* were detected in the follicle cells (*arrows*) and the terminal region of the rostrum (*yellow arrowhead*) [74]. Ubiquitous signals were detected with *APC*-*8904* sense riboprobes. **k** Illustration displaying presented developmental stages. In situ signals of *AAAP*-*536* and *APC*-*8904*, location of germ cells, *Buchnera*, and gut are indicated by the *color key*. *Scale bars* are all 20 μm. *bc Buchnera* symbionts, *cns* central nervous system, *g* gut, *gc* germ cells, *hd* head, *lg* legs, *st* stage

We detected the expression of *AAAP*-*536* and *APC*-*8904* mRNAs in oocytes and early embryos of asexual *A. pisum* using whole-mount in situ hybridization with DIG-labeled RNA probes. *AAAP*-*536* expression was not detected in the newly segregated oocyte (Fig. 1a), mature divided oocyte (Fig. 1b), syncytial embryos (Fig. 1c, d), or early blastoderm embryos (Fig. 1e). Expression of *AAAP*-*536* was first identified in the nuclei within the posterior syncytium of the stage 6 blastoderm embryos (Fig. 1f; Additional file 4: Figure S1). After stage 6, a maternal symbiotic bacterial mass invaded the embryos (developmental stage 7) from the posterior opening between the enlarged follicle cells. Strong expression of *AAAP*-*536* was identified in the region of the bacteria during the time that the bacterial mass was mixing with the posterior syncytial nuclei (Fig. 2a. To understand the relationship between the localization of syncytial nuclei and the bacterial mass, refer to Additional file 5: Figure S2 and note that panel g presents the same developmental stage as presented in Fig. 2a). Once most of the symbiotic bacterial mass has flowed to the embryo, gastrulation is initiated and germband invagination begins (developmental stages 8 and 9). At this point, we observed preferential expression of *AAAP*-*536* in the anterior region of the bacterial mass (where the posterior syncytial nuclei were located), and progressively weaker expression of *AAAP*-*536* in the posterior tail-like region of the bacterial mass (Fig. 2b, c, indicated with arrows). Unexpectedly, some transcripts of *AAAP*-*536* were detected in the periphery of nuclei outside of the bacterial mass after invasion of endosymbionts at developmental stages 8 and 9 (Fig. 2b, c, indicated with arrowheads). We infer that these *AAAP*-*536*-positive nuclei are the syncytial nuclei of the central syncytium and that later they are incorporated with the bacterial mass as bacteriocyte nuclei. *AAAP*-*536* signals remained in the bacterial region of the gastrula, from stage 8 to stage 10 (Fig. 2b–d). Later in development, at the time that the germband undergoes segmentation and limb bud formation, the bacterial mass compartmentalizes, with individual bacteriocyte cells becoming conspicuous (stages 10–14). At this time, transcripts of *AAAP*-*536* remained associated with the symbiotic bacterial mass (stages 11–14, Fig. 3a–d) and relatively strong signals of *AAAP*-*536* were found around the nuclei of bacteriocytes in segmented embryos at stage 11 and stage 12 (Fig. 3a, b, indicated with arrowheads). Strong expression of *AAAP*-*536* was also detected in embryonic bacteriocytes during limb bud formation (stages 13–14) (Fig. 3c, d). *AAAP*-*536* expression in bacteriocytes continued following completion of segmentation (stage 14) through katatrepsis (stage 15) (Fig. 3e). Following katatrepsis, bacteriocytes are grouped into the bilobed structure called the bacteriome. While *AAAP*-*536* expression was found primarily in the embryonic bacteriome (Fig. 4a–d), *AAAP*-*536* signals were additionally observed in the follicular epithelium at the egg chamber surface from stage 13 (Figs. 3c–e, 4b–d, indicated with arrows) and as non-specific signal in the terminal region of the rostrum in embryos after stage 18 (Fig. 4e, indicated with yellow arrowhead).

Unlike *AAAP*-*536*, we observed ubiquitous background signals of *APC*-*8904* in the germarium, the newly segregated oocyte (Fig. 1g), and mature divided oocyte (Fig. 1h). In syncytial embryos and early blastoderm embryos, transcripts of *APC*-*8904* were randomly distributed (Fig. 1i–k, indicated with arrowheads). Similar to *AAAP*-*536*, in late stage 6 blastoderm embryos, we observed preferential localization of *APC*-*8904* transcripts in the posterior syncytium (Fig. 1l); importantly, these preferential posterior syncytium *APC*-*8904* signals were not found in the sense control group (Fig. 1m, n). Similar to *AAAP*-*536*, we observed transcripts of *APC*-*8904* in the region occupied by the bacterial mass: during *Buchnera* invasion (Fig. 2e–g), bacteriocyte formation (stages 10–14) (Figs. 2h, 3f–i), and bacteriome maturation (stages 15–20) (Figs. 3j, 4f–i).

To summarize, we highlight the similarities and differences in expression of *AAAP*-*536* and *APC*-*8904* mRNAs. With respect to similarities, (1) preferential expression of both transporter genes was first recognized in nuclei of the posterior syncytium of the blastula (developmental stage 6 in Fig. 1f, l); (2) transcripts of both genes did not always colocalize with all *Buchnera* cells (Fig. 2b, c, f, g, h, indicated with arrows), but rather both appeared at the *Buchnera* migration front such that we observed colocalization of both transcripts and *Buchnera* in the anterior region of the bacterial mass (stages 8–9); and (3) signals for both transporters were detected during *Buchnera* invasion, bacteriocyte formation, and bacteriome maturation (Figs. 2, 3, 4). In contrast, where *AAAP*-*536* transcripts were detected in the periphery of nuclei outside the bacterial region during *Buchnera* invasion (Fig. 2b, c, indicated with arrowheads), transcripts of *APC*-*8904* were not (Fig. 2f, g).

### *AAAP*-*536* and *APC*-*8904* were expressed very early in development

**Fig. 5:**
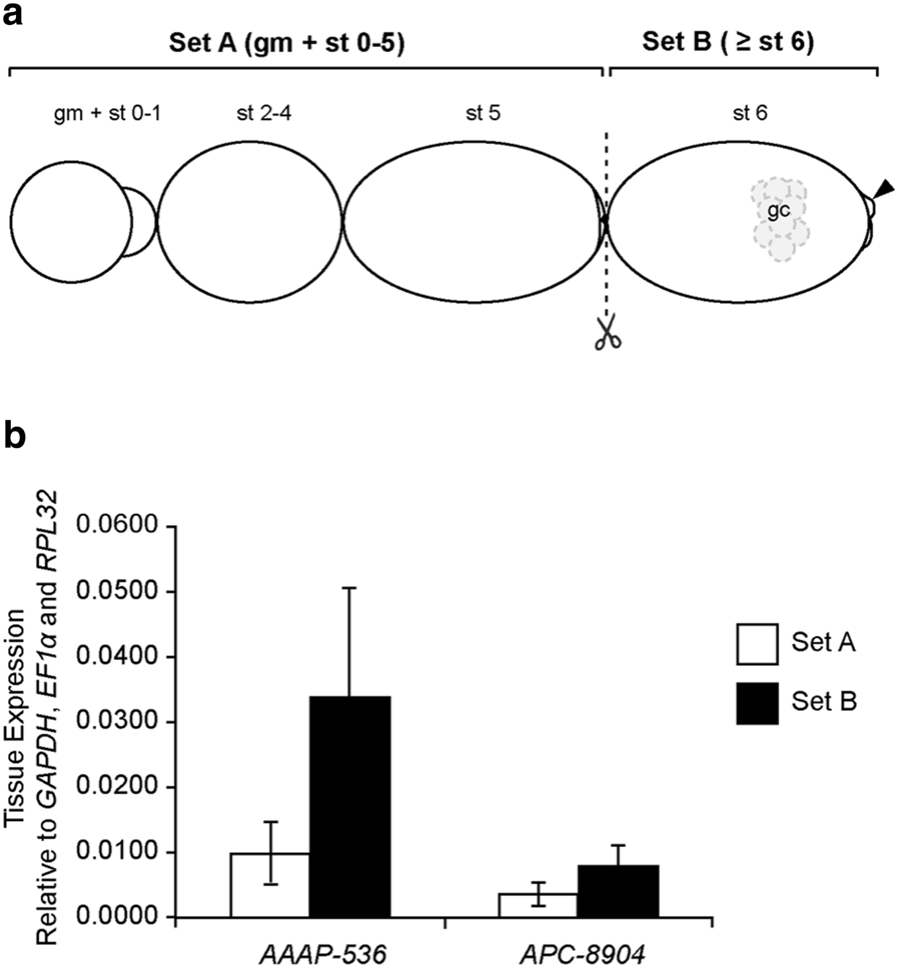
Relative expression of transporter genes in developing embryos before stage 5 and after stage 6. Comparison of *AAAP*-*536* and *APC*-*8904* expression between embryos before stage 5 and after stage 6 of development. **a** Outline of dissection. Set A: germaria, oocytes (st 0–2) and embryos at or before stage 5 of development; set B: embryos at or older than stage 6 of development. Set A and set B samples were separated by discriminating morphological recognizable germ cells (gc) and also the significantly enlarged follicle cells (*arrowhead*) in stage 6 embryos. **b** Results of quantitative reverse transcription real-time PCR. Expression of *AAAP*-*536* and *APC*-*8904* in aphid tissues normalized against three reference genes (*GAPDH*, *EF1α* and *RPL32*). *Bar heights* indicate mean of the gene expressions across three biological replicates, and *error bars* indicate 95% confidence interval estimates of the mean expression. Student *t* test comparisons of gene expression levels between embryos before stage 5 and embryos after stage 6 are not significant (*p* value = 0.26 in *AAAP*-*536* and *p* value = 0.32 in *APC*-*8904*). *gm* germaria, *gc* germ cells, *st* stage

We were intrigued by the expression of *AAAP*-*536* and *APC*-*8904* immediately prior to *Buchnera* invasion in the posterior syncytium of stage 6 embryos and wondered whether transcript detection so early in development was the result of maternal provisioning or zygotic expression. Thus, we further interrogated the start point of *AAAP*-*536* and *APC*-*8904* gene expression patterns in two ways. First, we reduced the stringency of in situ hybridization conditions for testing the earliest expression of *AAAP*-*536*—before stage 6—by decreasing the hybridization temperature to 60 °C in the hope that we would increase our ability to detect early signal if the control group remained clean under the reduced stringency hybridization condition. Unfortunately, reducing the stringency of the hybridization conditions resulted in detection of ubiquitous signals in the sense control group making it impossible to obtain more information regarding the expression of *AAAP*-*536* before stage 6 by in situ hybridization (Additional file 6: Figure S3). Given that relaxing the stringency of hybridization conditions did not facilitate obtaining greater insight, we then utilized quantitative reverse transcription real-time PCR, an approach that is more sensitive than in situ hybridization, to detect *AAAP*-*536* and *APC*-*8904* transcripts before stage 5 and after stage 6 of embryogenesis. While *AAAP*-*536* and *APC*-*8904* expression was high after stage 6, we found no significant difference in *AAAP*-*536* and *APC*-*8904* expression in embryos before stage 5 and after stage 6 (Fig. 5). This result was also repeated using a genetically discrete line of *A. pisum*, LSR1 (Additional file 7: Figure S4). Our results in both *A. pisum* lines demonstrated that *AAAP*-*536* and *APC*-*8904* were transcribed before stage 5 (Fig. 5 and Additional file 7: Fig. S4). Previously it has been argued that transcripts detected before stage 6 of development are of maternal origin, while those detected after stage 6 originate from the zygote [52, 53]. If this is the case, we infer that *AAAP*-*536* and *APC*-*8904* were maternally provisioned during early asexual aphid embryogenesis in *A. pisum*.

## Discussion

### Developmental integration of *Buchnera* into aphid hosts

Aphid work that targets elucidating the mechanisms of host/endosymbiont developmental integration has shown that: (1) specification of bacteriocytes involves a two-step determination process [17], (2) cellular mechanisms of *Buchnera* transmission from maternal bacteriocytes to embryos involve exocytotic and endocytotic cellular processes [16], and (3) maternal and embryonic bacteriomes are not equivalent [2, 54, 55]. Here, we extend work elucidating the mechanisms of host/endosymbiont developmental integration in aphids by describing the developmental atlas of *AAAP*-*536* and *APC*-*8904* mRNAs with respect to bacteriocyte specification and *Buchnera* transmission. In this study, we demonstrated that *AAAP*-*536* and *APC*-*8904* are expressed at the site of *Buchnera* invasion ahead of *Buchnera* transmission from mother to progeny (Fig. 1f, l, indicated with arrowheads). Following *Buchnera* invasion, we found that *AAAP*-*536* and *APC*-*8904* expression was not concomitant with the entirety of the bacterial mass, but rather expression of *AAAP*-*536* and *APC*-*8904* overlaps with only the anterior part of the bacterial mass in association with the syncytial nuclei (Fig. 2b, c, f–h). In addition, we typically observed signal from *AAAP*-*536* in the periphery of nuclei outside the bacterial mass (Fig. 2b, c, indicated with arrowheads); remarkably these nuclei localize to the areas that the bacterial mass moves to in the next developmental stage (Figs. 2b–d, 3a, b). Our results are consistent with the observations of Braendle et al. [17] and the hypothesis of Miura et al. [8] that the developmental origin of bacteriocytes involves a two-step determination process, the first from nuclei in the posterior syncytium and the second from the nuclei in the central syncytium. In contrast to Braendle et al. [17], who observed development of the second set of bacteriocytes at stage 13, our data suggest that recruitment of nuclei to the second set of bacteriocytes occurs as early as stage 8 of development (Fig. 2b, c). Alternatively, it is possible that *AAAP*-*536* and *Dll*, the marker used by Braendle et al. [17], are tracking the recruitment of two different sets of cells. By synthesizing the work of Braendle et al. [17] and Koga et al. [16] with the work we present here, we propose that the nuclei recruited at stage 8, defined by *AAAP*-*536* expression, are destined to form bacteriocyte cells, where the nuclei recruited at stages 13–15 are destined to form sheath cells (cells that are not occupied by *Buchnera* with nuclei much smaller than those of bacteriocytes [16, 17]).

While the clear and discrete expression of *AAAP*-*536* and *APC*-*8904* mRNA at the site of *Buchnera* invasion prior to *Buchnera* invasion suggests that both genes are involved in *Buchnera* recruitment to the blastula, the equally clear and discrete expression of *AAAP*-*536* and *APC*-*8904* in the posterior syncytium in association with the bacterial mass throughout embryogenesis suggests that both genes are involved additionally in bacteriocyte development and possibly play roles in regulating the transovarial transmission of *Buchnera*.

### Amino acid transporters *AAAP*-*536* and *APC*-*8904* do more than move amino acids across membranes

A handful of *Drosophila* orthologs of *AAAP*-*536* and *APC*-*8904* have been functionally characterized. On the basis of our observations of *AAAP*-*536* and *APC*-*8904* expression and the functional characterization of *Drosophila* orthologs, we hypothesize that *AAAP*-*536* and *APC*-*8904* function not to move amino acids across membranes but rather serve as effectors of the endocytotic transfer of *Buchnera* from maternal bacteriocytes to embryos and as nutrient sensors. *AAAP*-*536*, the most highly expressed bacteriocyte-expressed amino acid transporter, is orthologous to *Drosophila CG8785* [34], a gene whose functionally characterized paralogs include *pathetic* [24] and *polyphemus* (*CG12943*) [33]. Encoding a proton-assisted transporter (PAT) related to the mammalian solute carrier 36 (SLC36) amino acid transporters, *pathetic* is a well-known mediator of cell growth [24, 27, 31, 56]. Localized to the cell surface and late endosomes and lysosomes (LELs) of *Drosophila* fat body, *pathetic* senses extracellular amino acids and activates the target of rapamycin (TOR) signaling pathway [24, 29, 56]. The other functionally characterized *CG8785* paralog, *polyphemus*, encodes a glutamate transporter that functions to maintain immune-related function by modulating phagocytosis of microbial pathogens and controlling bacterial growth during infection [33]. The second most highly expressed bacteriocyte-expressed amino acid transporter, *APC*-*8904*, is orthologous to *Drosophila slimfast* [21, 34] and closely related to the *slimfast* paralogs *minidiscs* [30] and *genderblind* [57]. *Drosophila slimfast* and *minidiscs* functions are associated with nutrition and tissue growth involving the TOR signaling pathway [20, 21, 30, 58]. Encoding a glutamate transporter, *genderblind* functions in the nervous system with mutations in *genderblind* yielding a homosexual behavior phenotype [25, 57]. In addition to controlling glutamatergic synapse strength, *genderblind* functions with *polyphemus* to affect phagocytosis of microbial pathogens [33]. Significantly, taken together, functionally characterized *Drosophila* transporters related to *AAAP*-*536* and *APC*-*8904* are nutrient sensors that function as upstream mediators that activate the TOR signaling pathway [23]. Remarkably, activation of TOR signaling stimulates bulk endocytic uptake via clathrin-mediated endocytosis [26, 56]. In light of this genetic evidence from *Drosophila* and the expression of *AAAP*-*536* and *APC*-*8904* in the posterior syncytium, we hypothesize that *AAAP*-*536* and *APC*-*8904* gene products coordinate with the TOR signaling pathway to acquire *Buchnera* endosymbionts through an endocytic process that results in reacquisition of the symbiosomal membrane at the time of blastula invasion.

### An evolutionary perspective on bacteriocyte formation in insects

In contrast to the little that is known at a molecular level about the developmental specification of host bacteriocytes, other aspects of host/symbiont biology are becoming clear. It is clear that host/symbiont genome coevolution involves host/symbiont metabolic collaboration within metabolic pathways for amino acid [1, 6, 7, 54, 59–61] and vitamin biosynthesis [62]. It is also clear that while host/symbiont genome coevolution is constrained by host genome gene content [63–66], it can also involve the acquisition of new genes by lateral gene transfer [55, 63–65, 67, 68], and duplication of existing genes [34, 36–38, 69, 70]. While characterizing patterns of host/symbiont genome coevolution has been facilitated greatly by the recent accumulation of genome data from host/symbiont pairs, it has also been facilitated by studying bacteriocyte transcriptomes [36, 54, 59, 63, 71]. Bacteriocyte cells that house heritable endosymbionts are common among sap-feeding insects [72]. Substantial insights are likely to be gained by systematically studying similarities and differences in the genes and gene families expressed in bacteriocytes within a comparative framework. Further, which gene sets are conserved in their expression across a diversity of sap-feeding insects through development will likely facilitate determination of the functions that modulate development of the bacteriocyte.

The molecular mechanisms of bacteriocyte development were recently documented in the seed bug, *Nysius plebeius* [73]. Bacteriocyte cells of *N. plebeius* differentiate under regulation of homeobox transcription factors that include *Ultrabithorax* (*Ubx*), *abdominal*-*A* (*abd*-*A*), and *Antennapedia* (*Antp*). In gene knockdown experiments, suppression of *Ubx* results in the disappearance of bacteriocyte cells and failure of symbionts to localize, while suppression of *abd*-*A* alters the spatial organization of bacteriocyte cells, and suppression of *Antp* results in mislocalization of bacteriomes [73]. Notably, homeobox transcription factors that include Ubx/Abd-A, Distal-less (Dll), and Engrailed (En) are also expressed in aphid bacteriocytes [17]. While it is known that Dll is involved in aphid bacteriocyte determination [17], the way that Dll and other homeobox transcription factors determine bacteriocyte formation and bacteriome organization in aphids remains an open question.

## Conclusions

We are motivated to understand the metabolic and developmental integration of hosts and symbionts in order to understand host/symbiont coevolution. We selected the two amino acid transporters that are most highly expressed in *A. pisum* bacteriocytes and applied whole-mount in situ hybridization to analyze their developmental expression patterns. While we conjecture that *AAAP*-*536* and *APC*-*8904* gene products are integral to bacteriocyte development, we think that the most intriguing implication of this work concerns our inference that *AAAP*-*536* and *APC*-*8904* gene products modulate TOR signaling. Interpreting our data with knowledge that blastula acquire *Buchnera* by endocytosis [16] leads us to venture that *AAAP*-*536* and *APC*-*8904* stimulate the endocytotic process by coordinating with the TOR signaling pathway to faithfully facilitate the vertical transmission of *Buchnera* from mother to embryo across tens of millions of years of evolution. Notable, within an evolutionary framework, is the fact that the orthologs of *AAAP*-*536* and *APC*-*8904* show the same patterns of bacteriocyte-enriched expression in the green peach aphid, *Myzus persicae* [36]. Thus, we speculate that Mper-AAP11 and Mper-APC08 [36] are similarly expressed during asexual embryogenesis in *M. persicae*. This study highlights that duplicated amino acid transporters have potentially been retained in the aphid genome to play novel developmental roles in symbiont recruitment and genesis of the aphid bacteriome.

## Additional files

**Additional file 1: Table S1.** Sample numbers for the *AAAP*-*536* in situ hybridization experiments by developmental stage.

**Additional file 2: Table S2.** Sample numbers for the *APC*-*8904* in situ hybridization experiments by developmental stage.

**Additional file 3: Table S3.** Real-time PCR primers for *AAAP*-*536*, *APC*-*8904*, and control genes.

**Additional file 4: Figure S1.** DAPI counterstain of *A. pisum* asexual embryo at stage 6. The same stage 6 embryo as shown in Fig. 1f. Dashed lines indicate the location of the germ cells. (a) *AAAP*-*536* signal detected with NBT/BCIP brown precipitates and (b) DNA staining of the preparation in (a). No endosymbiotic bacterial DNA signals were detected, confirming that *AAAP*-*536* is expressed before *Buchnera* invasion. Scale bars are all 20 μm. Abbreviations: ps, posterior syncytium; eflc, enlarged follicle cells.

**Additional file 5: Figure S2.** Locations of *Buchnera* during asexual *A. pisum* embryogenesis. Confocal images of 19 developmental stages of asexual *A. pisum* embryogenesis are shown. DAPI staining labels *A. pisum* and symbiont *Buchnera* DNA with white. *Buchnera* cells are positioned in a pack and can be recognized by their round shape and 3 μm diameter. Green dashed lines mark the location of germ cells. Morphological characteristics of developmental stages are according to Miura et al. [8]. Scale bars are all 20 μm. Abbreviations: am, amnion; bc, *Buchnera* symbionts, cl, cephalic lobe; cns, central nervous system; cs, central syncytium; dn, dividing nucleus; eflc, enlarged follicle cells; fc, follicle cells; g, gut; gc, germ cells; gl, germarial lumen; gm, germarium; hd, head; ig, invaginating germband; lb, labial segment; lg, legs; mn, mandible segment; mx, maxilla segment; nc, nurse cells; o, oocyte; on, oocyte nucleus; pb, polar body; ps, posterior syncytium; s, serosa; sn, syncytial nucleus; st, stage; t1–3, thoracic segments.

**Additional file 6: Figure S3.** Whole-mount in situ hybridization of *AAAP*-*536* at 60 °C. Staged embryos hybridized with DIG-labeled antisense (a–f) and sense riboprobes (g-l) of *AAAP*-*536*. Signals were revealed by NBT/BCIP brown precipitates. All embryos are displayed with anterior of the germaria to the left. Arrowheads mark *AAAP*-*536*-positive signals in the syncytial nuclei. Dashed lines mark the location of germ cells. Arrow indicates *AAAP*-*536* signal not associated with *Buchnera*. Open arrowheads indicate signals detected in the follicle cells, and yellow arrowhead marks non-specific signal. For developmental staging, please refer to [8]. **(a, b)** Embryos before *Buchnera* invasion; transcripts of *AAAP*-*536* were detected in the posterior syncytium (ps) (arrowheads) in late stage 6 embryos. **(c–f)** *Buchnera* invasion starts at stage 7 and some *AAAP*-*536* transcripts were detected outside of the bacterial mass (arrowheads). After stage 11, *AAAP*-*536* transcripts were associated with the localization of endosymbiotic bacteria throughout development. **(g-l)** Developing embryos hybridized with sense riboprobes. Weak ubiquitous signals were detected in the embryos throughout development. Scale bars are all 20 μm. Abbreviations: bc, *Buchnera* symbionts; cns, central nervous system; gm, germarium; hd, head; ps, posterior syncytium; st, stage; t1–3, thoracic segments

**Additional file 7: Figure S4.** *AAAP*-*536* and *APC*-*8904* gene expression in two *A. pisum* lineages, NTU and LSR1. Comparison of *AAAP*-*536* and *APC*-*8904* expression between embryos before stage 5 and after stage 6 of development. Set A: germaria, oocytes (st 0–2), and embryos at or before stage 5 of development; set B: embryos at or older than stage 6 of development. Expression of *AAAP*-*536* and *APC*-*8904* in aphid tissues normalized to *GAPDH*. Bar heights indicate mean of the gene expressions across three biological replicates, and error bars indicate 95% confidence interval estimates of the mean expression. (a) Expression of *AAAP*-*536* and *APC*-*8904* in NTU; Student *t* test comparisons of gene expression levels between embryos before stage 5 and embryos after stage 6 are not significant (*p* value = 0.08 in *AAAP*-*536* and *p* value = 0.19 in *APC*-*8904*). (b) Data for LSR1 lineage were collected using the same sample preparation methods as NTU except that a qScript cDNA SuperMix (Quanta Biosciences) was used for cDNA synthesis, 1x PerfeCTa SYBR Green FastMix (Quanta Biosciences) was used for PCR products labeling, and Mastercycler ep realplex real-time PCR system (Eppendorf) was used for running samples. Expression of *AAAP*-*536* and *APC*-*8904* in LSR1 in embryos before stage 5 and embryos after stage 6 was not significantly different (Student *t* test: *p*-value = 0.07 for *AAAP*-*536* and *p*-value = 0.40 for *APC*-*8904*)

## Abbreviations

AAAP: amino acid/auxin permease family
*abd*-*A*: *abdominal*-*A*
*Antp*: *Antennapedia*
APC: amino acid/polyamine/organocation family
BCIP: 5-bromo-4-chloro-3-indolyl phosphate
*βTUB*: *β*-*tubulin*
bp: base pairs
DAPI: 4′,6-diamidino-2-phenylindole
DIG: digoxigenin
Dll: Distal-less
*EF1α*: *elongation factor 1*-*alpha*
En: engrailed
*GAPDH*: *glyceraldehyde*-*3*-*phosphate dehydrogenase*
h: hours
LELs: late endosomes and lysosomes
min: minute
NBT: nitroblue tetrazolium
ORF: open reading frame
PAT: proton-dependent amino acid transporter
PBS: phosphate-buffered saline
PCR: polymerase chain reaction
*RPL7*: *ribosomal protein L7*
*RPL32*: *ribosomal protein L32*
s: seconds
SLC36: mammalian solute carrier 36
TOR: target of rapamycin
*Ubx*: *Ultrabithorax*
UTR: untranslated region

## References

[1] Shigenobu S, Wilson ACC (2011). Genomic revelations of a mutualism: the pea aphid and its obligate bacterial symbiont. Cell Mol Life Sci.

[2] Lu HL, Price DRG, Wikramanayake A, Chang CC, Wilson ACC (2016). Ontogenetic differences in localization of glutamine transporter ApGLNT1 in the pea aphid demonstrate that mechanisms of host/symbiont integration are not similar in the maternal versus embryonic bacteriome. EvoDevo..

[3] Douglas AE (2006). Phloem-sap feeding by animals: problems and solutions. J Exp Bot.

[4] Karley AJ, Douglas AE, Parker WE (2002). Amino acid composition and nutritional quality of potato leaf phloem sap for aphids. J Exp Biol.

[5] Sandstrom J, Pettersson J (1994). Amino acid composition of phloem sap and the relation to intraspecific variation in pea aphid (*Acyrthosiphon pisum*) performance. J Insect Physiol.

[6] Russell CW, Poliakov A, Haribal M, Jander G, van Wijk KJ, Douglas AE (2014). Matching the supply of bacterial nutrients to the nutritional demand of the animal host. Proc Biol Sci..

[7] Wilson ACC, Ashton PD, Calevro F, Charles H, Colella S, Febvay G (2010). Genomic insight into the amino acid relations of the pea aphid, *Acyrthosiphon pisum*, with its symbiotic bacterium *Buchnera aphidicola*. Insect Mol Biol.

[8] Miura T, Braendle C, Shingleton A, Sisk G, Kambhampati S, Stern DL (2003). A comparison of parthenogenetic and sexual embryogenesis of the pea aphid *Acyrthosiphon pisum* (Hemiptera: Aphidoidea). J Exp Zool B Mol Dev Evol..

[9] Caillaud CM, Rahbe Y (1999). Aposymbiosis in a cereal aphid: reproductive failure and influence on plant utilization. Ecol Entomol..

[10] Douglas AE (2000). Reproductive diapause and the bacterial symbiosis in the sycamore aphid *Drepanosiphum platanoidis*. Ecol Entomol..

[11] Ishikawa H, Yamaji M (1985). Symbionin, an aphid endosymbiont-specific protein-I production of insects deficient in symbiont. Insect Biochem..

[12] Jayaraj S, Ehrhardt P, Schmutterer H (1967). The effect of certain antibiotics on reproduction of the black bean aphid, *Aphis fabae* Scop. Ann Appl Biol..

[13] Blackman RL, Minks AK, Harrewijn P (1987). Reproduction, cytogenetics and development. Aphids: their biology, natural enemies and control.

[14] Will L (1888). Entwicklungsgeschichte der Viviparen Aphiden.

[15] Blackman RL (1978). Early development of parthenogenetic egg in 3 species of aphids (Homoptera Aphididae). Int J Insect Morphol Embryol.

[16] Koga R, Meng XY, Tsuchida T, Fukatsu T (2012). Cellular mechanism for selective vertical transmission of an obligate insect symbiont at the bacteriocyte-embryo interface. Proc Natl Acad Sci U S A..

[17] Braendle C, Miura T, Bickel R, Shingleton AW, Kambhampati S, Stern DL (2003). Developmental origin and evolution of bacteriocytes in the aphid-*Buchnera* symbiosis. PLoS Biol.

[18] Fukatsu T, Nikoh N, Kawai R, Koga R (2000). The secondary endosymbiotic bacterium of the pea aphid *Acyrthosiphon pisum* (Insecta: homoptera). Appl Environ Microbiol.

[19] Griffiths GW, Beck SD (1973). Intracellular symbiotes of the pea aphid, *Acyrthosiphon pisum*. J Insect Physiol.

[20] Hundal HS, Taylor PM (2009). Amino acid transceptors: gate keepers of nutrient exchange and regulators of nutrient signaling. Am J Physiol Endocrinol Metab..

[21] Colombani J, Raisin S, Pantalacci S, Radimerski T, Montagne J, Leopold P (2003). A nutrient sensor mechanism controls *Drosophila* growth. Cell.

[22] Hyde R, Taylor PM, Hundal HS (2003). Amino acid transporters: roles in amino acid sensing and signalling in animal cells. Biochem J.

[23] Taylor PM (2014). Role of amino acid transporters in amino acid sensing. Am J Clin Nutr.

[24] Goberdhan DCI, Meredith D, Richard Boyd CA, Wilson C (2005). PAT-related amino acid transporters regulate growth via a novel mechanism that does not require bulk transport of amino acids. Development..

[25] Grosjean Y, Grillet M, Augustin H, Ferveur JF, Featherstone DE (2008). A glial amino-acid transporter controls synapse strength and courtship in *Drosophila*. Nat Neurosci.

[26] Hennig KM, Colombani J, Neufeld TP (2006). TOR coordinates bulk and targeted endocytosis in the *Drosophila melanogaster* fat body to regulate cell growth. J Cell Biol.

[27] Lin WY, Williams C, Yan C, Koledachkina T, Luedke K, Dalton J (2015). The SLC36 transporter Pathetic is required for extreme dendrite growth in *Drosophila* sensory neurons. Genes Dev.

[28] Armstrong AR, Laws KM, Drummond-Barbosa D (2014). Adipocyte amino acid sensing controls adult germline stem cell number via the amino acid response pathway and independently of target of rapamycin signaling in *Drosophila*. Development..

[29] Heublein S, Kazi S, Ogmundsdottir MH, Attwood EV, Kala S, Richard Boyd CA (2010). Proton-assisted amino-acid transporters are conserved regulators of proliferation and amino-acid-dependent mTORC1 activation. Oncogene.

[30] Martin JF, Hersperger E, Simcox A, Shearn A (2000). *minidiscs* encodes a putative amino acid transporter subunit required non-autonomously for imaginal cell proliferation. Mech Dev.

[31] Lin WY, Williams CR, Yan C, Parrish JZ (2015). Functions of the SLC36 transporter Pathetic in growth control. Fly..

[32] Boll M, Daniel H, Gasnier B (2004). The SLC36 family: proton-coupled transporters for the absorption of selected amino acids from extracellular and intracellular proteolysis. Pflugers Arch.

[33] Gonzalez EA, Garg A, Tang J, Nazario-Toole AE, Wu LP (2013). A glutamate-dependent redox system in blood cells is integral for phagocytosis in *Drosophila melanogaster*. Curr Biol.

[34] Price DRG, Duncan RP, Shigenobu S, Wilson ACC (2011). Genome expansion and differential expression of amino acid transporters at the aphid/*Buchnera* symbiotic interface. Mol Biol Evol.

[35] Price DRG, Feng H, Baker JD, Bavan S, Luetje CW, Wilson ACC (2014). Aphid amino acid transporter regulates glutamine supply to intracellular bacterial symbionts. Proc Natl Acad Sci U S A..

[36] Duncan RP, Feng H, Nguyen DM, Wilson ACC (2016). Gene family expansions in aphids maintained by endosymbiotic and non-symbiotic traits. Genome Biol Evol..

[37] Dahan RA, Duncan RP, Wilson ACC, Davalos LM (2015). Amino acid transporter expansions associated with the evolution of obligate endosymbiosis in sap-feeding insects (Hemiptera: sternorrhyncha). BMC Evol Biol.

[38] Duncan RP, Husnik F, Van Leuven JT, Gilbert DG, Davalos LM, McCutcheon JP, Wilson ACC (2014). Dynamic recruitment of amino acid transporters to the insect/symbiont interface. Mol Ecol.

[39] AphidBase: an aphid genomics database. INRA, Rennes. 2016. http://bipaa.genouest.org/is/aphidbase/. Accessed 28 Oct 2016.

[40] The International Aphid Genomics Consortium (2010). Genome sequence of the pea aphid *Acyrthosiphon pisum*. PLoS Biol.

[41] Chang CC, Lee WC, Cook CE, Lin GW, Chang T (2006). Germ-plasm specification and germline development in the parthenogenetic pea aphid *Acyrthosiphon pisum*: Vasa and Nanos as markers. Int J Dev Biol.

[42] Chang CC, Lin GW, Cook CE, Horng SB, Lee HJ, Huang TY (2007). *Apvasa* marks germ-cell migration in the parthenogenetic pea aphid *Acyrthosiphon pisum* (Hemiptera: Aphidoidea). Dev Genes Evol.

[43] Chang CC, Huang TY, Shih CL, Lin GW, Chang TP, Chiu H, Chang WC (2008). Whole-mount identification of gene transcripts in aphids: protocols and evaluation of probe accessibility. Arch Insect Biochem Physiol.

[44] Hellemans J, Mortier G, De Paepe A, Speleman F, Vandesompele J (2007). qBase relative quantification framework and software for management and automated analysis of real-time quantitative PCR data. Genome Biol.

[45] Vandesompele J, De Preter K, Pattyn F, Poppe B, Van Roy N, De Paepe A, Speleman F. Accurate normalization of real-time quantitative RT-PCR data by geometric averaging of multiple internal control genes. Genome Biol. 2002;3:RESEARCH0034. 10.1186/gb-2002-3-7-research0034 PMC126239 12184808

[46] Andersen CL, Jensen JL, Orntoft TF (2004). Normalization of real-time quantitative reverse transcription-PCR data: a model-based variance estimation approach to identify genes suited for normalization, applied to bladder and colon cancer data sets. Cancer Res.

[47] Pfaffl MW, Tichopad A, Prgomet C, Neuvians TP (2004). Determination of stable housekeeping genes, differentially regulated target genes and sample integrity: BestKeeper–Excel-based tool using pair-wise correlations. Biotechnol Lett.

[48] Ishikawa A, Ogawa K, Gotoh H, Walsh TK, Tagu D, Brisson JA (2012). Juvenile hormone titre and related gene expression during the change of reproductive modes in the pea aphid. Insect Mol Biol.

[49] Whyard S, Singh AD, Wong S (2009). Ingested double-stranded RNAs can act as species-specific insecticides. Insect Biochem Mol Biol.

[50] Shakesby AJ, Wallace IS, Isaacs HV, Pritchard J, Roberts DM, Douglas AE (2009). A water-specific aquaporin involved in aphid osmoregulation. Insect Biochem Mol Biol.

[51] Livak KJ, Schmittgen TD (2001). Analysis of relative gene expression data using real-time quantitative PCR and the 2(-Delta Delta C(T)) Method. Methods.

[52] Chang CC, Hsiao YM, Huang TY, Cook CE, Shigenobu S, Chang TH (2013). Noncanonical expression of *caudal* during early embryogenesis in the pea aphid *Acyrthosiphon pisum*: maternal *cad*-driven posterior development is not conserved. Insect Mol Biol.

[53] Huang TY, Cook CE, Davis GK, Shigenobu S, Chen RPY, Chang CC (2010). Anterior development in the parthenogenetic and viviparous form of the pea aphid, *Acyrthosiphon pisum*: *hunchback* and *orthodenticle* expression. Insect Mol Biol.

[54] Hansen AK, Degnan PH (2014). Widespread expression of conserved small RNAs in small symbiont genomes. ISME J.

[55] Nakabachi A, Ishida K, Hongoh Y, Ohkuma M, Miyagishima SY (2014). Aphid gene of bacterial origin encodes a protein transported to an obligate endosymbiont. Curr Biol.

[56] Ogmundsdottir MH, Heublein S, Kazi S, Reynolds B, Visvalingam SM, Shaw MK, Goberdhan DCI (2012). Proton-assisted amino acid transporter PAT1 complexes with Rag GTPases and activates TORC1 on late endosomal and lysosomal membranes. PLoS ONE.

[57] Augustin H, Grosjean Y, Chen K, Sheng Q, Featherstone DE (2007). Nonvesicular release of glutamate by glial xCT transporters suppresses glutamate receptor clustering *in vivo*. J Neurosci.

[58] Reynolds B, Roversi P, Laynes R, Kazi S, Richard Boyd CA, Goberdhan DCI (2009). *Drosophila* expresses a CD98 transporter with an evolutionarily conserved structure and amino acid-transport properties. Biochem J.

[59] Hansen AK, Moran NA (2011). Aphid genome expression reveals host-symbiont cooperation in the production of amino acids. Proc Natl Acad Sci U S A..

[60] Rabatel A, Febvay G, Gaget K, Duport G, Baa-Puyoulet P, Sapountzis P (2013). Tyrosine pathway regulation is host-mediated in the pea aphid symbiosis during late embryonic and early larval development. BMC Genom.

[61] Russell CW, Bouvaine S, Newell PD, Douglas AE (2013). Shared metabolic pathways in a coevolved insect-bacterial symbiosis. Appl Environ Microbiol.

[62] Price DRG, Wilson ACC (2014). A substrate ambiguous enzyme facilitates genome reduction in an intracellular symbiont. BMC Biol.

[63] Husnik F, Nikoh N, Koga R, Ross L, Duncan RP, Fujie M (2013). Horizontal gene transfer from diverse bacteria to an insect genome enables a tripartite nested mealybug symbiosis. Cell.

[64] Moran NA, Jarvik T (2010). Lateral transfer of genes from fungi underlies carotenoid production in aphids. Science.

[65] Sloan DB, Nakabachi A, Richards S, Qu J, Murali SC, Gibbs RA, Moran NA (2014). Parallel histories of horizontal gene transfer facilitated extreme reduction of endosymbiont genomes in sap-feeding insects. Mol Biol Evol.

[66] Wilson ACC, Duncan RP (2015). Signatures of host/symbiont genome coevolution in insect nutritional endosymbioses. Proc Natl Acad Sci U S A..

[67] Nikoh N, McCutcheon JP, Kudo T, Miyagishima SY, Moran NA, Nakabachi A (2010). Bacterial genes in the aphid genome: absence of functional gene transfer from *Buchnera* to its host. PLoS Genet.

[68] Nikoh N, Nakabachi A (2009). Aphids acquired symbiotic genes via lateral gene transfer. BMC Biol.

[69] Duncan RP, Nathanson L, Wilson ACC (2011). Novel male-biased expression in paralogs of the aphid *slimfast* nutrient amino acid transporter expansion. BMC Evol Biol.

[70] Shigenobu S, Stern DL (2013). Aphids evolved novel secreted proteins for symbiosis with bacterial endosymbiont. Proc Biol Sci..

[71] Nakabachi A, Shigenobu S, Sakazume N, Shiraki T, Hayashizaki Y, Carninci P (2005). Transcriptome analysis of the aphid bacteriocyte, the symbiotic host cell that harbors an endocellular mutualistic bacterium, *Buchnera*. Proc Natl Acad Sci U S A..

[72] Douglas AE (1989). Mycetocyte symbiosis in insects. Biol Rev Camb Philos Soc.

[73] Matsuura Y, Kikuchi Y, Miura T, Fukatsu T (2015). *Ultrabithorax* is essential for bacteriocyte development. Proc Natl Acad Sci U S A..

[74] Chang CC, Huang TY, Cook CE, Lin GW, Shih CL, Chen RPY (2009). Developmental expression of *Apnanos* during oogenesis and embryogenesis in the parthenogenetic pea aphid *Acyrthosiphon pisum*. Int J Dev Biol.

