# Supplementary material for: Amino acid transporters implicated in endocytosis of Buchnera during symbiont transmission in the pea aphid
Source: EvoDevo. 2016 Nov 21;7:24. doi: 10.1186/s13227-016-0061-7 (PMC5117694; doi:10.1186/s13227-016-0061-7)
Supplement: Supplementary file 1 — Additional file 1: Table S1. Sample numbers for the AAAP-536 in situ hybridization experiments by developmental stage. [file 13227_2016_61_MOESM1_ESM.docx]

**Additional file 1: Table S1. Sample numbers for the *AAAP-536* *in situ* hybridization experiments by developmental stage**.

| Stage | Signal | No. of antisense samples | No. of sense samples | Positive signal (%) |
| --- | --- | --- | --- | --- |
| gm |  | 32 | 11 | - |
| 0 |  | 7 | 2 | - |
| 1 |  | 8 | 3 | - |
| 2 |  | 1 | 2 | - |
| 3 |  | 3 | 4 | - |
| 4 |  | 4 | 1 | - |
| 5 |  | 4 | 1 | - |
| 6 | ps | 22 | 9 | 12/22 (54.55%) |
| 7 | bc | 12 | 4 | 12/12 (100%) |
| 8 | bc | 5 | 1 | 5/5 (100%) |
| 9 | bc | 4 | 0 | 4/4 (100%) |
| 10 | bc | 8 | 0 | 8/8 (100%) |
| 11 | bc | 4 | 0 | 4/4 (100%) |
| 12 | bc | 7 | 1 | 7/7 (100%) |
| 13 | bc | 7 | 1 | 7/7 (100%) |
| 14 | bc | 11 | 0 | 11/11 (100%) |
| 15 | bc | 9 | 0 | 9/9 (100%) |
| 16 | bc | 1 | 0 | 1/1 (100%) |
| 17 | bc | 2 | 0 | 2/2 (100%) |
| 18 | bc | 1 | 1 | 1/1 (100%) |
| 19 | bc | 1 | 0 | 1/1 (100%) |

bc, endosymbiotic symbiont; gm, germaria; ps, posterior syncytium.
