# Supplementary material for: Amino acid transporters implicated in endocytosis of Buchnera during symbiont transmission in the pea aphid
Source: EvoDevo. 2016 Nov 21;7:24. doi: 10.1186/s13227-016-0061-7 (PMC5117694; doi:10.1186/s13227-016-0061-7)
Supplement: Supplementary file 3 — Additional file 3: Table S3. Real-time PCR primers for AAAP-536, APC-8904, and control genes. [file 13227_2016_61_MOESM3_ESM.docx]

**Additional file 3: Table S3. Real-time PCR primers for *AAAP-536*, *APC-8904*, and control genes.**

| **Gene** | **Gene ID** | **Forward primer (5' - 3')** | **Reverse primer (5' - 3')** | **Amplicon** | **Efficiency** | **R^2^** | **Reference** |
| --- | --- | --- | --- | --- | --- | --- | --- |
| **Amino Acid Transporters** | | | | | | | |
| *AAAP-536* | *ACYPI000536* | TCTTTGCCATGGAGGGTATC | CGGAGGAAATGTTCTGGATG | 73 bp | 107% | 0.98991 | [34] |
| *APC-8904* | *ACYPI008904* | TGCTGTTCAGCTTTGTGACC | TTGGAAGGCCGATGAGTTAC | 83 bp | 105% | 0.99060 | [34] |
| **Control Genes** | | | | | | | |
| *GAPDH* | *ACYPI009769* | TGGTGTTGAGAGCTTCGTTG | GCCTTTGAAACGTCCATGAG | 122 bp | 104% | 0.99182 | [34] |
| *EF1α* | *ACYPI006711*  *ACYPI004434* | GAACACGCTCTATTGGCTTTCAC | CCATCTTGTTCACACCAACGA | 64 bp | 108% | 0.98809 | [48] |
| *RPL7* | *ACYPI010200* | TTGAAGAGCGTAAGGGAACTG | TATTGGTGATTGGAATGCGTTG | 76 bp | 104% | 0.99324 | [49] |
| *RPL32* | *ACYPI000074* | CGTCTTCGGACTCTGTTGTCAA | CAAAGTGATCGTTATGACAAACTCAA | 74 bp | 96% | 0.99237 | [50] |
| *βTUB* | *ACYPI001007* | GGCCAAGGGTCATTACACTGA | TGCGAACCACGTCCAACA | 62 bp | 101% | 0.99232 | [50] |
