# Supplementary figures and images for: Amino acid transporters implicated in endocytosis of Buchnera during symbiont transmission in the pea aphid
Source: EvoDevo. 2016 Nov 21;7:24. doi: 10.1186/s13227-016-0061-7 (PMC5117694; doi:10.1186/s13227-016-0061-7)

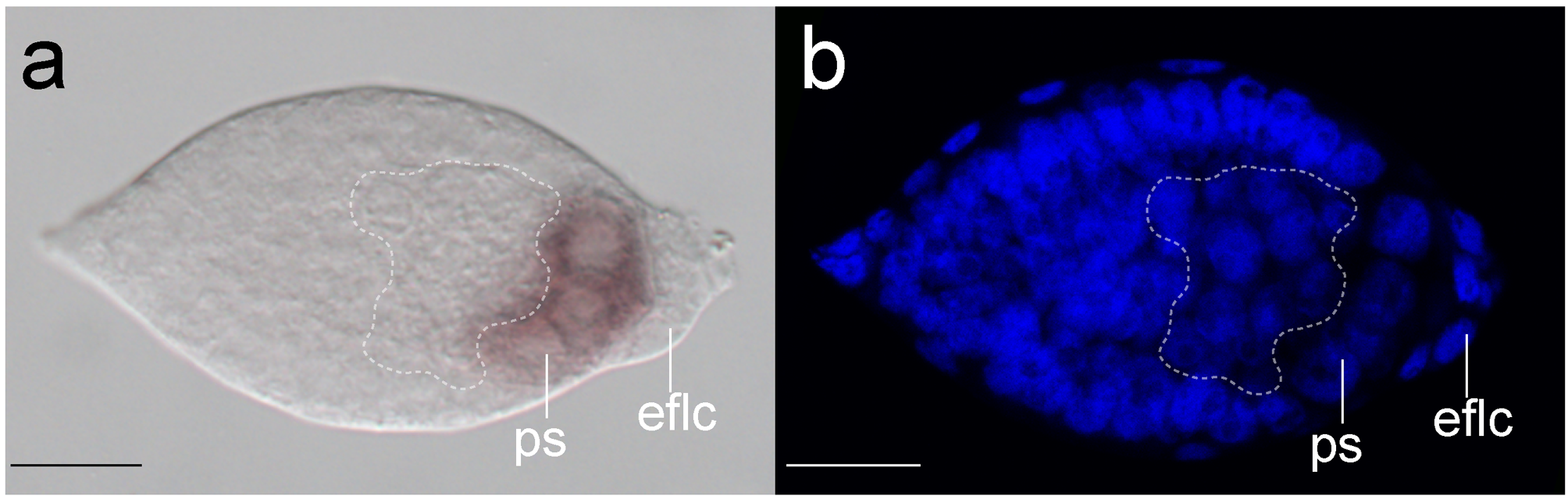

Supplement: Supplementary file 4 — Additional file 4: Figure S1. DAPI counterstain of A. pisum asexual embryo at stage 6. The same stage 6 embryo as shown in Fig. 1f. Dashed lines indicate the location of the germ cells. (a) AAAP-536 signal detected with NBT/BCIP brown precipitates and (b) DNA staining of the preparation in (a). No endosymbiotic bacterial DNA signals were detected, confirming that AAAP-536 is expressed before Buchnera invasion. Scale bars are all 20 μm. Abbreviations: ps, posterior syncytium; eflc, enlarged follicle cells. [file 13227_2016_61_MOESM4_ESM.tif]

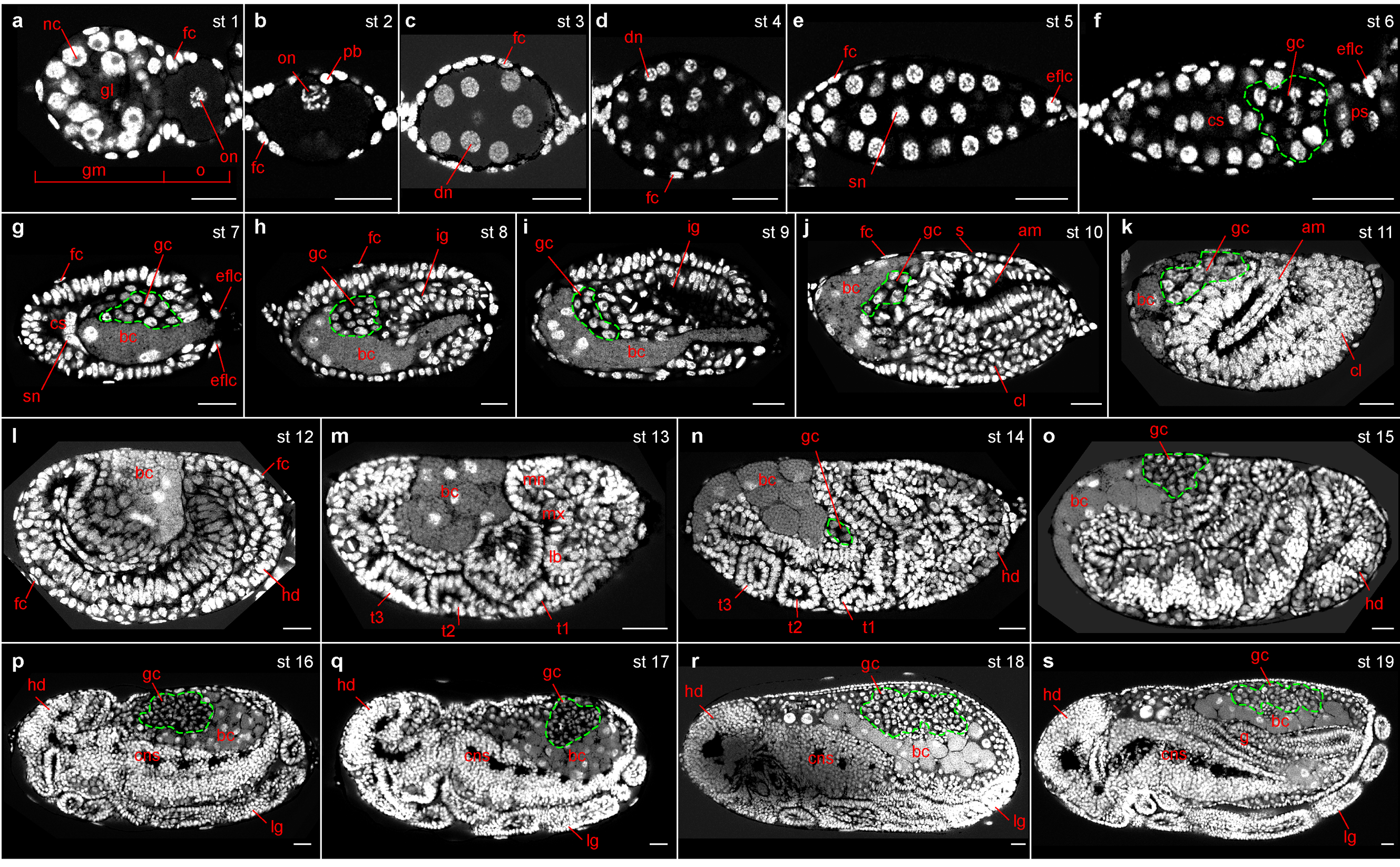

Supplement: Supplementary file 5 — Additional file 5: Figure S2. Locations of Buchnera during asexual A. pisum embryogenesis. Confocal images of 19 developmental stages of asexual A. pisum embryogenesis are shown. DAPI staining labels A. pisum and symbiont Buchnera DNA with white. Buchnera cells are positioned in a pack and can be recognized by their round shape and 3 μm diameter. Green dashed lines mark the location of germ cells. Morphological characteristics of developmental stages are according to Miura et al. [8]. Scale bars are all 20 μm. Abbreviations: am, amnion; bc, Buchnera symbionts, cl, cephalic lobe; cns, central nervous system; cs, central syncytium; dn, dividing nucleus; eflc, enlarged follicle cells; fc, follicle cells; g, gut; gc, germ cells; gl, germarial lumen; gm, germarium; hd, head; ig, invaginating germband; lb, labial segment; lg, legs; mn, mandible segment; mx, maxilla segment; nc, nurse cells; o, oocyte; on, oocyte nucleus; pb, polar body; ps, posterior syncytium; s, serosa; sn, syncytial nucleus; st, stage; t1–3, thoracic segments. [file 13227_2016_61_MOESM5_ESM.tif]

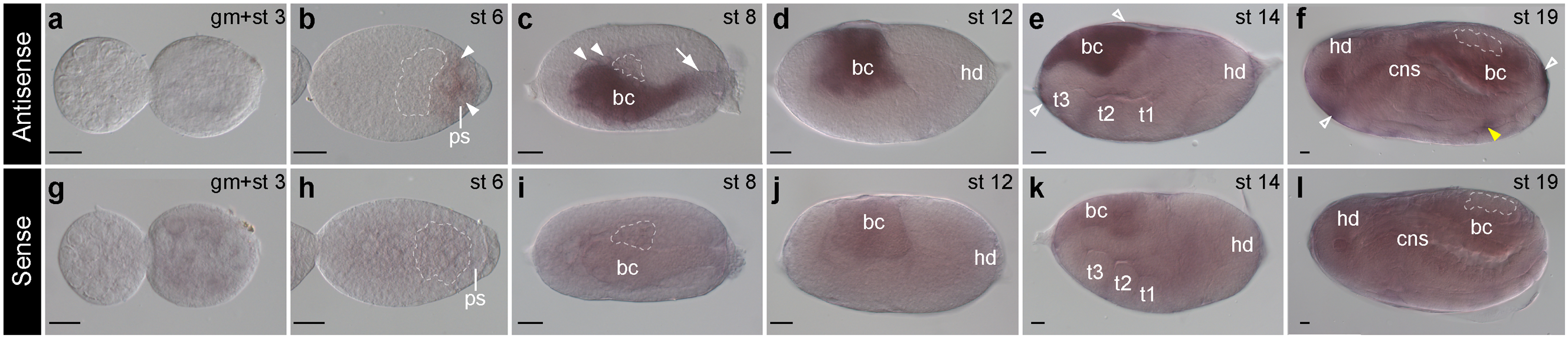

Supplement: Supplementary file 6 — Additional file 6: Figure S3. Whole-mount in situ hybridization of AAAP-536 at 60 °C. Staged embryos hybridized with DIG-labeled antisense (a–f) and sense riboprobes (g-l) of AAAP-536. Signals were revealed by NBT/BCIP brown precipitates. All embryos are displayed with anterior of the germaria to the left. Arrowheads mark AAAP-536-positive signals in the syncytial nuclei. Dashed lines mark the location of germ cells. Arrow indicates AAAP-536 signal not associated with Buchnera. Open arrowheads indicate signals detected in the follicle cells, and yellow arrowhead marks non-specific signal. For developmental staging, please refer to [8]. (a, b) Embryos before Buchnera invasion; transcripts of AAAP-536 were detected in the posterior syncytium (ps) (arrowheads) in late stage 6 embryos. (c–f) Buchnera invasion starts at stage 7 and some AAAP-536 transcripts were detected outside of the bacterial mass (arrowheads). After stage 11, AAAP-536 transcripts were associated with the localization of endosymbiotic bacteria throughout development. (g-l) Developing embryos hybridized with sense riboprobes. Weak ubiquitous signals were detected in the embryos throughout development. Scale bars are all 20 μm. Abbreviations: bc, Buchnera symbionts; cns, central nervous system; gm, germarium; hd, head; ps, posterior syncytium; st, stage; t1–3, thoracic segments [file 13227_2016_61_MOESM6_ESM.tif]

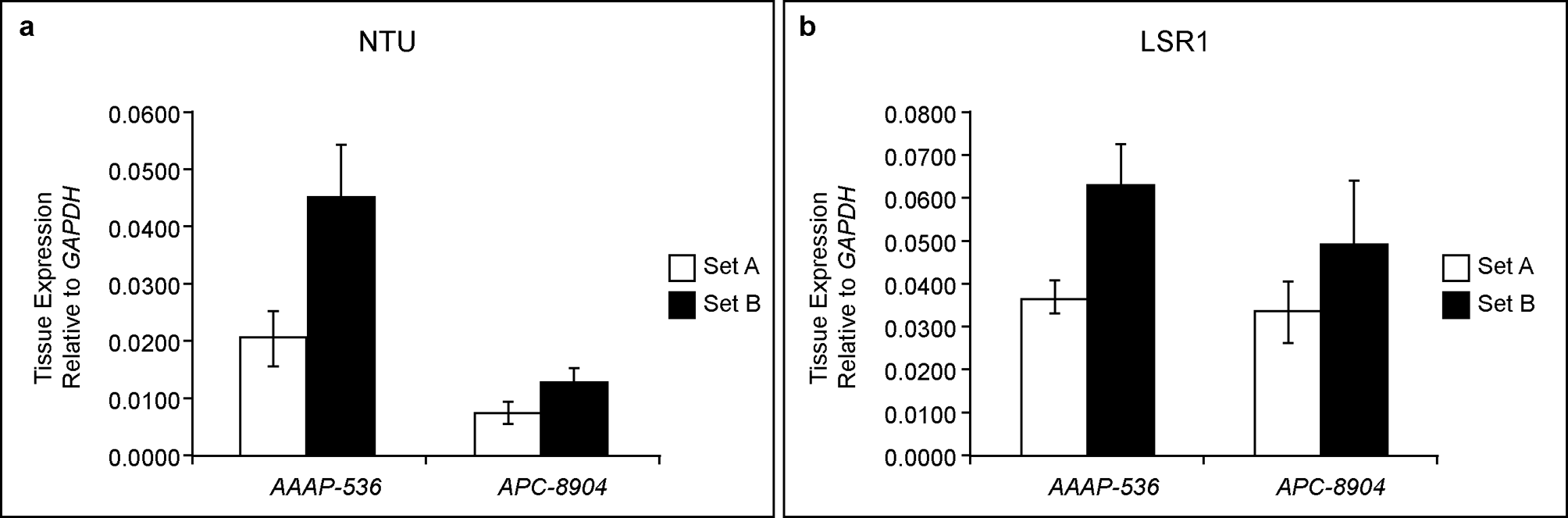

Supplement: Supplementary file 7 — Additional file 7: Figure S4. AAAP-536 and APC-8904 gene expression in two A. pisum lineages, NTU and LSR1. Comparison of AAAP-536 and APC-8904 expression between embryos before stage 5 and after stage 6 of development. Set A: germaria, oocytes (st 0–2), and embryos at or before stage 5 of development; set B: embryos at or older than stage 6 of development. Expression of AAAP-536 and APC-8904 in aphid tissues normalized to GAPDH. Bar heights indicate mean of the gene expressions across three biological replicates, and error bars indicate 95% confidence interval estimates of the mean expression. (a) Expression of AAAP-536 and APC-8904 in NTU; Student t test comparisons of gene expression levels between embryos before stage 5 and embryos after stage 6 are not significant (p value = 0.08 in AAAP-536 and p value = 0.19 in APC-8904). (b) Data for LSR1 lineage were collected using the same sample preparation methods as NTU except that a qScript cDNA SuperMix (Quanta Biosciences) was used for cDNA synthesis, 1x PerfeCTa SYBR Green FastMix (Quanta Biosciences) was used for PCR products labeling, and Mastercycler ep realplex real-time PCR system (Eppendorf) was used for running samples. Expression of AAAP-536 and APC-8904 in LSR1 in embryos before stage 5 and embryos after stage 6 was not significantly different (Student t test: p-value = 0.07 for AAAP-536 and p-value = 0.40 for APC-8904) [file 13227_2016_61_MOESM7_ESM.tif]
